# American Sign Language Syntax and Analogical Reasoning Skills Are Influenced by Early Acquisition and Age of Entry to Signing Schools for the Deaf

**DOI:** 10.3389/fpsyg.2016.01982

**Published:** 2016-12-26

**Authors:** Jon Henner, Catherine L. Caldwell-Harris, Rama Novogrodsky, Robert Hoffmeister

**Affiliations:** ^1^Professions in Deafness, Department of Specialized Education Services, University of North Carolina at GreensboroGreensboro, NC, USA; ^2^Department of Psychological and Brain Sciences, Boston UniversityBoston, MA, USA; ^3^Department of Communication Sciences and Disorders, University of HaifaHaifa, Israel; ^4^Programs in Deaf Studies, Center for the Study of Communication and the Deaf, Boston UniversityBoston, MA, USA

**Keywords:** ASL acquisition, deaf children, analogies, syntax, age of acquisition

## Abstract

Failing to acquire language in early childhood because of language deprivation is a rare and exceptional event, except in one population. Deaf children who grow up without access to indirect language through listening, speech-reading, or sign language experience language deprivation. Studies of Deaf adults have revealed that late acquisition of sign language is associated with lasting deficits. However, much remains unknown about language deprivation in Deaf children, allowing myths and misunderstandings regarding sign language to flourish. To fill this gap, we examined signing ability in a large naturalistic sample of Deaf children attending schools for the Deaf where American Sign Language (ASL) is used by peers and teachers. Ability in ASL was measured using a syntactic judgment test and language-based analogical reasoning test, which are two sub-tests of the ASL Assessment Inventory. The influence of two age-related variables were examined: whether or not ASL was acquired from birth in the home from one or more Deaf parents, and the age of entry to the school for the Deaf. Note that for non-native signers, this latter variable is often the age of first systematic exposure to ASL. Both of these types of age-dependent language experiences influenced subsequent signing ability. Scores on the two tasks declined with increasing age of school entry. The influence of age of starting school was not linear. Test scores were generally lower for Deaf children who entered the school of assessment after the age of 12. The positive influence of signing from birth was found for students at all ages tested (7;6–18;5 years old) and for children of all age-of-entry groupings. Our results reflect a continuum of outcomes which show that experience with language is a continuous variable that is sensitive to maturational age.

## Introduction

Studying language deprivation in Deaf children has led to important findings on brain plasticity and sensitive periods in human development (Corina and Singleton, 2009). The temporal lobes, important for processing and understanding auditory language, are activated by sign language in congenitally Deaf subjects, indicating striking neural plasticity (Nishimura et al., 1999). In the realm of language acquisition, the topic of this paper, studying Deaf individuals with late acquisition of a first language has helped quantify the notion of sensitive periods for language learning (e.g., Mayberry and Eichen, 1991; Mayberry, 1993). Mayberry and colleagues have shown across two decades of research that outcomes for learning a first language, and any subsequent languages, are progressively worse for individuals with later age-of-acquisition (Mayberry and Lock, 2003; Boudreault and Mayberry, 2006; Mayberry, 2010).

The notion of “windows for language and cognitive development” that are briefly opened and then closed continues to be debated by scientists, educators, and the public. The keen interest in this topic has also engendered myths and misunderstandings, alongside genuine unknowns (e.g., Giraud and Lee, 2007). To set the stage for presenting our data on a large naturalistic sample of over 600 school-aged Deaf children, we discuss two claims about the timing of acquisition of sign language that are based on little or no data but used to buttress advice to parents of Deaf children.

### (1) Time Windows for ASL Acquisition Close Too Soon

Knoors and Marschark (2012) claimed that the hearing parents of Deaf children may not be able to learn ASL well enough, or quickly enough, for their children to benefit from sign language used in the home. They cite data on sensitive periods for language acquisition, including the studies we cited above, of delayed language acquisition of ASL by Mayberry and Lock (2003) and Mayberry (2010). They further asserted that children of hearing parents are exposed to insufficient sign language at home during early and middle childhood, the time when the brain has maximal plasticity for learning a first language (Mayberry and Lock, 2003). Given this advice, hearing parents may hesitate to enroll their Deaf children in an academic signing environment, either as part of an early intervention program, or a preschool. They may infer that little is to be gained by learning sign language themselves, if they cannot become fluent quickly enough, or attain a high enough level soon enough, to facilitate their children’s language and cognitive development.

### (2) Can Sign Language Be the Back-Up Communication Method If Speech Training Fails?

Many medical, speech, and language therapists, audiological, and early intervention professionals recommend to parents that they not teach their Deaf children sign language lest it interfere with the acquisition of spoken language (although there is considerable diversity of option among practitioners, see Mellon et al., 2015). But, parents are sometimes told they can consider sign language as a back-up language in case speech therapies do not work (see Mauldin, 2016 for more information). Underlying these recommendations is the findings from neuroimaging studies that visual processing can “take over” neural regions that mediate hearing and spoken language in children (Nishimura et al., 1999). However, research indicates sign language acquisition does not interfere with spoken language acquisition (Hassanzadeh, 2012; Davidson et al., 2014). Indeed, there is emerging evidence that sign language may actually facilitate spoken language development and that it may counter the cognitive effects of language deprivation (Davidson et al., 2014; Amraei et al., 2017). The aspect of this debate addressed by our data concerns the advisability of using sign language as a *back-up* system.

It is noteworthy that these two claims give opposite advice to hearing parents of Deaf children. The first one tells hearing parents that sign language cannot be learned past a critical period, so do not bother placing your child in a school that uses ASL. The second claim advises parents that they can always switch to sign-language later in childhood, thus implying that maturational constraints on learning sign-language are minimal. Critical to this discussion is the fact that knowing a language fluently is a major factor in learning about the world and crucial for success in an academic environment. Additionally, for Deaf children, research supports the notion that fluency in a first language (e.g., ASL) supports learning a second language (Novogrodsky et al., 2014a). In fact, several studies have found that ASL knowledge support the development of English literacy skills (Lange et al., 2013; Andrew et al., 2014; Ausbrooks et al., 2014; Hrastinski and Wilbur, 2016). Hrastinski and Wilbur (2016) examined the relationship between ASL abilities and performance on several different standardized assessments, including the Stanford Achievement Test 10th edition, and the Measures of Academic Progress and their associated sub-tests. ASL proficiency was the most significant predictor of performance on the different assessments; more important than home language, whether or not the child was implanted, whether or not they had a speech or language impairment, or how old they were during assessment.

Important to both these claims are the actual ASL skills of Deaf children who vary in their entry to an academic signing environment. Our data addresses this, together with the comparison between signing from birth with Deaf parents vs. absent or unsystematic signing with hearing parents. As further background for our study, we review below what is known about language development in Deaf children.

## Language Development in Deaf Children

Language deprivation occurs when Deaf children are not exposed to sign language from birth and gain minimal information from spoken language. Delays in language milestones are typically observed in non-native signers and late-signers, broadly conceptualized as systematic exposure after age 5–7. The negative effects of late linguistic exposure are present in either the signed or spoken language modalities (Mayberry, 2010). Late exposure has far-reaching consequences. The most important are cognitive and social impairments which then compound difficulties adjusting to the mainstream classroom and larger society (e.g., Branson and Miller, 1993; Ramsey, 1997; Nunes et al., 2001). Late exposure often leads to behavioral problems, mental illness, and substance abuse (Black and Glickman, 2006; Glickman, 2007; Anderson et al., 2015).

In contrast to Deaf children with language deprivation, children acquiring sign language as a first language (L1) from birth pass through the same language acquisition stages and achieve the same cognitive milestones as do children acquiring spoken language as their first language (Newport and Meier, 1985; Petitto, 1987; Goldin-Meadow and Mylander, 1998; Mayberry and Squires, 2006; Corina and Singleton, 2009; Novogrodsky et al., 2014b). The primary barrier to acquiring sign language as a first language is that 95% Deaf children are born to hearing, non-signing parents, who most frequently use only spoken language (Mitchell and Karchmer, 2005). According to survey research from 2009 to 2010, 5.8% of hearing parents in the US reported using ASL (Gallaudet Reseach Institute, 2011).

Today, with the focus on early intervention and cochlear implants, many Deaf children have increased access to spoken language. But implants do not give sufficient support for spoken language acquisition to be successful for all Deaf children (Mellon et al., 2015). While attrition rates for implant users vary and appear to be low (Watson and Gregory, 2005; Archibold et al., 2009), not all children can use implants well enough to acquire spoken language to levels characteristic of their hearing peers (Niparko et al., 2010; Geers and Sedey, 2011; Geers et al., 2015). Implanted children also have poorer executive function abilities than typically developing children (Figueras et al., 2008; Kronenberger et al., 2013). They do not seem to acquire the same kind of language-based reasoning skills as hearing children (Edwards et al., 2011). We cite these findings not to disparage cochlear implants, but to remind readers that cochlear implants do not fully remediate language deprivation in Deaf children.

There is also now considerable evidence that learning sign language does not interfere with learning spoken language. Deaf children can be bi-modal bilinguals, as shown in these two research studies:

• Deaf children of Deaf signing parents, exposed to both a full natural sign language (ASL) from birth and spoken English after receiving cochlear implants (Davidson et al., 2014).
• Deaf children in early intervention programs who received both auditory/oral therapy and weekly sign language instruction from a fluent ASL user (Yoshinaga-Itano et al., 2010).

Children in these studies were able to acquire both sign language and spoken language without conflict. They demonstrated language performance within the normal age-range in both modalities (Davidson et al., 2014). These bi-modal children preformed at monolingual English age-targets on standardized language tests, including the Preschool Language Scale test (Zimmerman et al., 2002) and the Expressive Vocabulary Test (Williams, 2007). Receiving natural language input via the visual modality apparently minimized the negative effects of early auditory deprivation (Davidson et al., 2014). This allowed spoken language acquisition to be acquired in a time frame that is later than standard first language acquisition. This indicates the positive effects that are possible following early intervention.

Language development in Deaf children is tied to the development of language based analogical reasoning skills. Edwards et al. (2011) argued that historically low performance of Deaf children on analogical reasoning assessments, including language based analogical reasoning, may be related to language deprivation. Bandurski and Galkowski (2004), studied this in a sample of Deaf children who were native signers of Polish-sign language. When given an analogical reasoning assessment using polish-sign language, native signers performed on par with typically developing hearing children who were given an equivalent assessment in written Polish. Henner (2016), in his dissertation, demonstrated that the best predictor of performance on an ASL language based analogical reasoning assessment was ASL vocabulary ability, thereby building on the work of Bandurski and Galkowski (2004).

## The Effect of Age of Acquisition on Spoken and Sign Language Development of Deaf Children

As mentioned briefly above, the most compelling evidence regarding maturational constraints on first language learning has relied on late first language learners who are usually Deaf (e.g., Newport, 1990; Pénicaud et al., 2013). Newport (1990) discussed the ASL abilities of 30 adults, aged 35–70 years, who had a minimum of 30 years’ daily exposure to ASL, but differed in the age of first exposure to ASL. All participants were near ceiling in their understanding of basic ASL word order. Late learners (those exposed to sign language after age 12) had difficulties with morphology. They produced frozen signs, omitted obligatory morphemes, and were inconsistent on test items requiring the same morpheme. Early learners (who entered the school for the Deaf between ages 4–6) also had lower scores on tests of morphology than those who were exposed to ASL from birth from Deaf parents, indicating the importance of early and consistent ASL exposure at home.

Age of acquisition effects on language development have been more extensively studied by Mayberry and colleagues (Mayberry and Fischer, 1989; Mayberry and Lock, 2003; Boudreault and Mayberry, 2006; Mayberry and Squires, 2006; Mayberry, 2007). Different aspects of sign language have been studied: syntactic acquisition (Boudreault and Mayberry, 2006), narrative comprehension (Mayberry and Fischer, 1989), sentence memory (Mayberry and Fischer, 1989), sentence interpretation (Mayberry and Lock, 2003), and on-line grammatical processing (Mayberry and Eichen, 1991). These studies revealed that age of acquisition has long-lasting effects that are observable even when learners were adults who were tested after years of sign language experience (see also Mayberry, 1992).

The general public often learns about late first language acquisition through the exceptional case study of Genie (Curtiss, 1977), which involved extreme neglect and physical abuse. It remains unappreciated by the public at large that language deprivation among Deaf students continues to be common, even the norm. Language deprivation can occur in stable, loving families who work to provide their children with language.

It may also not be well known among parents that early intervention programs exist to support Deaf children’s acquisition of language, both signed and spoken. Hearing parents can expose their Deaf children to sign language by enrolling them into schools for the Deaf, where both peers and teachers use ASL. First exposure to ASL for hearing children is thus frequently the age of entrance to a school for the Deaf. Age of entry to a school for the Deaf is widely used in psycholinguistics to index of age of acquisition (Newport, 1990; Mayberry and Eichen, 1991; Mayberry, 1993; Mayberry et al., 2002, 2011; Henner et al., 2015; Novogrodsky et al., under review).

Age-of-entry to a school for Deaf varies greatly; there are several reasons why this is the case. The current policy in special education is for Deaf children to attend their local public school (mainstreaming) (e.g., Ramsey, 1997). Thus, when students transfer into a school for the Deaf, they have often transferred from either a non-signing school program or a non-ASL program. An example of a non-ASL program is one that uses an artificial signing system, like Signed Exact English (SEE). Such transfers often occur when students have failed academically in the mainstream education environment, typically due to problems caused by language deprivation. The majority of Deaf children of hearing parents appear to transfer into schools for the Deaf after the age of 6 (Henner et al., 2015).

In the current study we examine age effects in acquiring the different domains of syntax and vocabulary-based analogical reasoning. Prior work on age-effects in syntactic acquisition found difficulties when ASL was acquired between age 5–7, and even stronger difficulties when age of acquisition was between 8 and 13 (Boudreault and Mayberry, 2006). Boudreault and Mayberry studied adults; we attempt to replicate and extend those findings by testing school age Deaf children. Vocabulary-based analogical reasoning is also highly dependent on language skills and would also be affected by late age-of-acquisition (Richland and Burchinal, 2013). It is noteworthy that previous research (Sharpe, 1985) argued that auditory stimulation is necessary to develop language-based analogical reasoning skills. We examine these issues via a large sample of Deaf children growing up as visual learners, with ASL as the primary language.

## Overview of Method

As reviewed above, substantial literature exists on the importance of early exposure to signed language^1^ for later language development. We wanted to extend these findings to a large cohort of school-aged Deaf children (ages 7;6–18;5), and to ask more detailed questions about long-term outcomes of early signed language exposure, and, in the more specific case, ASL. Is experience with ASL from parents at birth more important for syntactic acquisition compared to acquisition of vocabulary? Deaf children frequently enter a school for the Deaf, where ASL is used in an academic context, at different ages. Does the age of entry to a school for the Deaf influence later ASL ability? Which of these factors, early experience with the language at home with parents, vs. systematic exposure to ASL in a school setting, is more important?

Our questions required participants with different histories of exposure to ASL. This required a larger sample than has been common in prior research with Deaf students. We were able to test the largest sample of Deaf children in the United States by collaborating with Deaf schools. This was also a necessity because the current project was part of our team’s larger goal of developing the American Sign Language Assessment Instrument (ASLAI), a computerized inventory of sign language assessments (Hoffmeister et al., unpublished). As part of a funded project by the US Institute of Education Sciences running from 2010 to 2015, and with agreements with the schools for the Deaf where we tested, we were able to secure “blanket consent” through the Boston University Institutional Review Board. Blanket consent meant that parents needed to opt their children out of assessment rather than opt in. In exchange for blanket consent, schools were provided with detailed reports about their students’ ASL abilities for use in individualized education program (IEP) planning. Blanket consent allowed us access to large and varied numbers of Deaf children.

The schools we targeted were residential schools for the Deaf with at least 100 or more students. These schools typically have relatively high numbers of Deaf teachers and staff, including native ASL fluent adults who provide high-quality visual language input. Schools for the Deaf tend to be favored among Deaf parents. These schools use ASL as the medium of academic instruction and thus have high levels of ASL classroom use in the classroom. Residential schools also have a large ASL-using peer population. Students thus learn ASL naturalistically via immersion, during ASL-mediated activities after school or during free time with both peers and adults. These schools have strong early intervention programs, meaning ASL exposure can begin in infancy. Given these characteristics, the environment of the residential schools facilitates both natural language acquisition and provides exposure to academic language in the classroom. One of our goals is to determine how much students can benefit from these sign-rich environments even if age of entry to the school for the Deaf occurs later than early childhood, during the elementary school years (age 6–12).

To make testing manageable at diverse schools around the country, we focused on collecting information about the students from the schools, and did not additionally survey parents. Schools for the Deaf record how many Deaf parents are at home, the year students entered the school, and other background information. We relied on these records and thus do not have information about the amount of signing at home, nor do we have key information about socio-economic status. We also do not know about any sign language exposure prior to entry into the school of assessment.

Relying on information provided by schools has the benefit that we have generally the same information for all students. It obviates the drawback of missing and subjective data that is customary with reliance on parental surveys. School records on whether students had Deaf parents allowed us to categorize those students as being native signers, meaning exposed to sign from birth. We categorized students with hearing parents as non-native signers, meaning that exposure to ASL before entry into the school of assessment was likely either absent or erratic in nature (Mitchell and Karchmer, 2005). Our primary variables were thus: (1) Native/non-native signing, and (2) Age of entry to the current school of assessment. Although these are imperfect measures of our underlying theoretical constructs (see below), they proved to be powerful predictors of students’ language outcomes, with effects that were measurable well into the teen years.

Our variables, operationalizations, and predictions were as follows:

### Native vs. Non-native Signing Status

Operationalized by having at least one Deaf vs. hearing parent, this variable captures the theoretical construct of early/systematic exposure to language vs. late/uneven exposure. We predicted better ASL for native signers, and predicted that this advantage would hold across all ages tested (i.e., from the onset of schooling until age 18;5, the age of ending high school).

### Age of Entry to School of Assessment

This was operationalized as the year that students entered the school where they were tested. This is frequently students’ first exposure to a consistent signing school environment that includes peers with signing abilities of equal or better skills. For a substantial majority of non-native students, date of entry to the current school for the Deaf represents the first systematic exposure to a signed language, and in some cases their first systematic exposure to any accessible language. It is thus related to the theoretical construct of age of first language acquisition. Note that the classic studies of the influence of late exposure to sign language used date of entry to a residential school for the Deaf as the onset of acquisition of ASL (see Henner et al., 2015 for review). However, when interpreting the variable of date of entry to the school for the Deaf, it is important to bear in mind that our population includes students with Deaf parents (native signers). For native signers, date of entry to the current school signals the onset of classroom ASL and the challenges and rigors that academic language presents compared to conversational language (Cummins, 1982).

Given that age of entry to school is related to first systematic exposure to ASL for non-native signers, we predicted that younger age of entry to school would correspond to higher scores on the two ASLAI tasks. This reflects the widespread idea in the language acquisition literature of “the younger the better” (see Singleton and Ryan, 2004). However, there are two caveats which make age of entry to school an exploratory variable. First, some students will have attended a signing school before the current school. For this reason, age of entry to the current school may overestimate that real average age of first exposure to signed language. It also reduces the opportunity to find that a late date of school entry is associated with poorer language outcomes. The second problem goes in the reverse direction. Late entry to a school of the Deaf is often a response to low academic achievement in a mainstream school setting. These students thus enroll at their first signing school not simply with advanced age, but with a history of poor language and academic failure. This will increase the association between late age and poor ASL ability.

## Materials and Methods

### Participants

Data for this study came from 688 Deaf students between the ages of 7;6 and 18;5 years from schools for the Deaf in the US who scored above chance (25%) on the tasks used to collect data (**Table 1**). The students attended the large schools mentioned above, which are considered “signing schools.” That is, they use sign language as the primary language of instruction. The students were administered the American Sign Language Assessment Instrument (ASLAI; Hoffmeister et al., unpublished). Two of the ASLAI tasks were analyzed for this study: a language based analogical reasoning task that represents vocabulary and grammatical knowledge and requires metalinguistic skills (Henner, 2016), and a syntactic judgment task representing ASL syntactic knowledge (Novogrodsky et al., under review). While all 688 students were tested on the analogy task, only 455 took the more recently developed syntactic judgment task. Parental hearing status was used as an approximate indicator of exposure to signed language from birth. The label “native” was given to participants who had at least one Deaf parent. The rationale for this categorization is that 92% of families with two Deaf parents use ASL at home and 84% of families with one Deaf parent use ASL at home (Mitchell and Karchmer, 2005). We thus considered 244 students to be native signers. As noted earlier, many of the remaining 444 non-native signers may not have been exposed to ASL until they entered the school for the Deaf where they were tested. Although the native signing group was smaller than the non-native signing group, they composed 35% of the sample population of this study. This is a significant representative sample considering it is estimated that only 5–10% of Deaf children are born to Deaf parents (Mitchell and Karchmer, 2005).

**Table 1:** Number of students by task, age, and signing status.

Age	8	9	10	11	12	13	14	15	16	17	18
**Analogy**											
Native	18	24	37	33	19	28	30	20	11	14	10
Non-native	25	35	39	40	33	39	53	43	47	41	49
**Syntax**											
Native	10	16	22	31	16	19	27	13	9	6	9
Non-native	13	25	21	20	21	25	40	23	36	27	26

**FIGURE 1:**
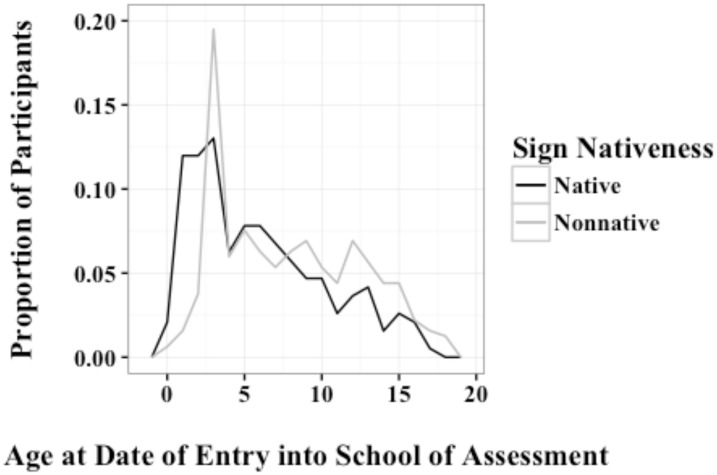
**Proportion of native and non-native students at the age at which they entered the school for the Deaf**.

An important variable for our analyses was the age at which students entered the school of assessment, as a proxy for the likely onset of systematic ASL exposure. For native signers, age of school entry indicates the onset of exposure to classroom ASL (i.e., academic ASL), which is significant given that schooling extends and enriches language abilities even for native speakers (e.g., Cummins, 1982). Three groupings were made based on the age of entry to the school of assessment. These groupings were 0–6 (early intervention programs to kindergarten/1st grade), 7–12 (elementary school), and 13–18 (post-elementary school). **Figure 1** shows the proportion of the frequency count for the different ages at which native and non-native students entered the schools for the Deaf.

A higher percentage of native signers entered a signing school for the Deaf by age six compared to non-native signers (63% vs. 52%). For date of entry between age 7–12, the percentages were 27 and 32%, respectively, and for entry after age 12, 9 and 16%. The sample of non-native signers in this study, compared to the US population of non-native Deaf signers, likely includes a higher percentage who entered a school for the Deaf before the age of six. Nevertheless, one can see that age of entry into a signing school is generally earlier for students with Deaf parents than those with hearing parents. It is worth noting that our sample also contained a large number of *native* signers who entered schools for the Deaf after the age of 6. Because we do not have information about students prior to entering the school of assessment, we cannot describe the exact reasons for this placement. Some of these students may have attended a different Deaf school prior to the school of assessment. Just like hearing parents, Deaf parents may have tried mainstreaming, oralism, or other educational options before deciding to place their child in a traditional school for the Deaf.

### Materials

The measures used in this study were two subtasks of the ASL Assessment Instrument. The ASLAI is a comprehensive battery of 11 multiple choice receptive language tasks presented via computer. Video presentation of stimuli and responses vary according to the task. The ASLAI platform is capable of displaying both pictures and videos. For most tasks, a stimulus is displayed, followed by four videos which are presented sequentially. Participants can interact with the videos to pause or replay them. Responses are selected by clicking a button above a corresponding video (e.g., A–D). While the test order was randomized, the question order was fixed for each participant. It is comparable in scope and content to spoken language tests administered to school-aged children. For assessing ASL syntactic knowledge, the Syntax Difficult subtest (for comparison, there is a Syntax Simple subtest) was used; for assessing vocabulary and related metalinguistic skills, the Analogy subtest was used. Here we briefly review the specific design of each task (see also Novogrodsky et al., under review).

#### (a) The Syntax Task

This syntactic judgment task includes 27 test items designed to tap knowledge of nine syntactic ASL structures (roughly following Boudreault and Mayberry, 2006): (a) Topicalization, (b) Subject-Verb-Object (SVO), (c) Complements, (d) Relative Clauses, (e) Verb agreement, (f) Negation, (g) Conditionals, (h) Wh-Q, and (i) Rhetorical Questions. The Syntax test in the ASLAI was modeled after Boudreault and Mayberry’s assessment, but it differs in crucial ways. First, the assessment developed by Boudreault and Mayberry had 168 sentences that functioned as individual questions (stimuli). These questions were either grammatical or not grammatical. In the ASLAI, a question was composed of four different videos: a stimulus item containing a grammatically correct sentence and three foils containing different syntactic violations. Glosses of a sample complement structure question are presented in 1a–d below. 1a is a gloss of the correct response, 1b and 1c are foils with word order violations, and 1d is a foil with incorrect co-indexing between FRIEND_j_ and HE_k_ representing a grammatical violation. Items varied in types of foils, such as word order, or incorrect non-manual markers.

(1a) Correct response: MY FRIEND HE_i_ THINK WE HAVE TEST TOMORROW.
(1b) Word order violation: TEST TOMORROW THINK WE HAVE MY FRIEND HE_*j*._
(1c) Word order violation: TOMORROW MY FRIEND HE_j_ HAVE THINK TEST.
(1d) Syntactic violation: MY FRIEND_i_ HE_j_ THINK WE HAVE TEST TOMORROW

Second, Boudreault and Mayberry (2006)’s assessment was designed for adults and their sample was composed of 30 Deaf adults. The Syntax test in the ASLAI was designed for children.

#### (b) The Analogy Task

It is an ASL analogical reasoning assessment. Items were presented using the classical sentence presentation, A : B :: C : ?. Students viewed a signed sentence in which the A, B, and C parts were signed, followed by the ASL sign for WHAT (five handshape, both hands). They then were presented with four signed responses, of which one was the intended target. The task was composed of 24 different questions that corresponded to one of six different types of analogies: (a) causality, (b) antonym (opposites), (c) part-whole, (d) ASL phonology, (e) purpose, and (f) noun-verb pairs (a derivational process common in ASL that changes a verb to a noun through reduplication).

### Testing Procedures

Participants completed the Analogies and Syntax tasks in groups of up to 20 students, with typical group sizes ranging from 5 to 20. The ASLAI computer platform presented the two tasks in four different phases using only ASL via video windows. We describe the three phases of each of the two tasks here.

#### Instructions

Students viewed instructions for each of the two tasks, presented in ASL in the actual task, but translated into English for ease of reading in this paper. The instructions for the syntax task were as followed: “Now you will take a different test. This test is a syntax test. You will see four different video windows presenting signed sentences. The format will be the same as the previous tests you have taken, with four separate movie windows on your screen. Each movie window will play one sentence. You need to carefully watch each sentence and decide which one of the four sentences is correct. Three of the sentences are wrong – the signing is wrong/incorrect and the way the signs fit together do not make sense. One sentence is right – it is produced correctly, the sign order is correct, facial expressions are correct, and everything fits together in the right way. You need to pick that one correct sentence.”

The instructions for the analogy task are as follows: “Now you will take an analogies test. What’s an analogies test? First you’ll see three signs. Two, A and B, have a relationship. The third sign C has the same relationship with D as A and B. Where is D? You have to find it from the four signs shown. How will you decide which one is the right one? Well, the first two signs A : B have a relationship and you will use that relationship to find the correct sign for C :.”

#### Practice

Before each task, students viewed practice items and were provided feedback on whether or not their selection was correct. Students were instructed to select the response that best reflected a correct response in ASL.

#### Task Procedures

In the Analogies task, for each of the 24 items, the testing platform presented a signed stimulus item that included an item that was missing the response in the second part of the analogy equation, this was followed with four videos containing one target sign and three foil signs. In the syntax task for each of the 27 items, the testing platform presented an instructional sentence for the stimulus (CHOOSE CORRECT ASL SENTENCE), and four video stimuli, which contained one target sentence and three foil sentences. Upon completion of each task a review screen appeared allowing students to go over their choices and make corrections before the final choice was made. In the review screen, the target item was displayed along with the freeze frame of the student’s selected response. Students could also return to a selected item and confirm their selection or select a different response if they choose.

## Results

### Hypothesis Testing With Mixed Linear Effects Models

Our goal was to determine the effects of early language experience with ASL on subsequent ASL skills by measuring outcomes during the school years. Effects in two domains were investigated: ASL syntactic judgment and language-based analogical reasoning. We were interested in two ways to measure early language experience:

• the age-of-entry into an academic signing environment (meaning enrolling at a school for the Deaf where ASL was used by teachers and peers)
• being a native vs. non-native signer (and thus having systematic exposure to ASL from birth vs. later, non-systematic exposure)

We were further interested in which one of these types of early language experience was more important for subsequent ASL outcomes.

Our analysis was accomplished using mixed linear effects models, with model fit measured by maximum likelihood estimations (MLL; Baayen et al., 2008). Statistical significance of the predictors was calculated by using chi-square to compare a new MLL value to the prior MLL value without that predictor in the model.

#### Age of Entry Into the School of Assessment

Age was included as a fixed effect to control for age-based abilities. We first ran the analysis with only age as a fixed effect, and test takers (students) as a random effect. We then added age at date of entry (as a fixed effect) and assessed whether this resulted in a significant change in the maximum likelihood estimation. Adding age at date of entry did improve the model fit for both syntactic judgment [χ^2^(5) = 33.95, *p* < 0.001], and language based analogies [χ^2^(5) = 30.65, *p* < 0.001]. In both models, the students accounted for roughly 2% of the variance (see summary in **Table 2**).

**Table 2:** Mixed effects multiple regression with age-of-entry as a predictor.

	*Syntactic Judgment*			*Analogies*		
	Estimate (Beta)	Standard Error	*t*-value	Estimate (Beta)	Standard Error	*t*-value
**Age**	0.03	0.003	8.55	0.03	0.003	10.30
**Age-at-Date-of-Entry**	–0.01	0.002	–5.94	–0.01	0.002	–5.60

The data in **Table 2** provide verification that increasing age-of-entry to school is associated with lower scores on both the syntactic and analogy tasks. The beta values shown in **Table 2** are the same for the two tasks, with the –0.01 value indicating that age-of-entry is a weak predictor. We suspected this was because the relationship between age-of-entry and the dependent variables was not linear. In the next analysis, age-of-entry to school was grouped into three categories, as described in the methods section. This allowed us to examine the decline in syntactic judgment and language based analogical reasoning skills with increasing age of school entry for specific age-of-entry periods. We could thus determine more precisely, where in childhood the maximum decline occurred and how strong it was. In the next analysis, we also added in native vs. non-native signing status.

#### Native vs. Non-native Signing Status

Because we changed age at date of entry into a grouping variable with three levels, we first calculated their predictive strength, without the presence of native vs. non-native signing status. We then added native vs. non-native as a predictor in the model (see statistical summary in **Table 3**).

**Table 3:** Mixed effects multiple regression with predictors age-of-entry and signing status.

	*Syntactic judgment*			*Analogies*		
	Estimate (Beta)	Standard Error	*t*-value	Estimate (Beta)	Standard Error	*t*-value
**Age**	0.03	0.003	9.42	0.03	0.003	8.20
**Age-at-Date -Of-Entry (0–6)**						
7–12	–0.04	0.02	–2.07	–0.03	0.02	–1.84
13–18	–0.13	0.03	–4.83	–0.13	0.03	–4.90
**Parental Signing Status (Native)**						
Non-native	–0.12	0.02	–6.90	–0.13	0.02	–7.70

*^1^By signed language, we mean a fully operationalized language, such as ASL rather than a sign system invented to model a spoken language through signs, such as Signed Exact English (SEE)*.

Age at date of entry was a significant predictor for both syntactic judgment [χ^2^(7) = 81.08, *p* < 0.001] and analogical reasoning [χ^2^(7) = 117.59, *p* < 0.001]. The age-at-date-of-entry groups were significant for syntactic judgment [χ^2^(7) = 23.14, *p* < 0.001] and analogical reasoning [χ^2^(7) = 23.52, *p* < 0.001]. Native vs. non-native status was also significant for syntactic judgment abilities [χ^2^(7) = 45.18, *p* < 0.001] and analogical reasoning skills [χ^2^(7) = 56.45, *p* < 0.001]. Again, students themselves accounted for 2% of the variance.

The decrease in test scores for those entering the school for the Deaf after age 12 was especially drastic, as can be seen by the relatively large beta weights of -0.13. Non-native signers on average scored 12 and 13 points lower on the syntactic judgment task and analogical reasoning tasks compared to native signers. The results highlight the importance of early language experience on syntactic sensitivity and language based analogical reasoning skills.

### Visualizing the Influence of Signing Status on ASL Outcomes

**FIGURE 2:**
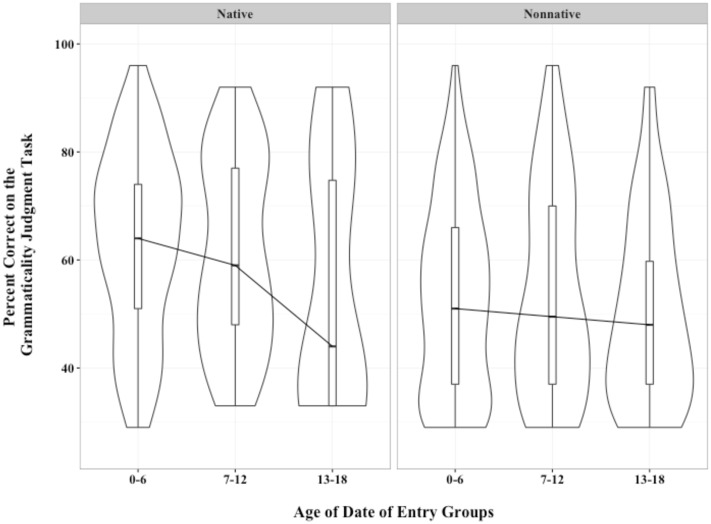
**Density plots showing percent correct on the syntactic judgment task for native and non-native signers by age of date of entry groups**.

**FIGURE 3:**
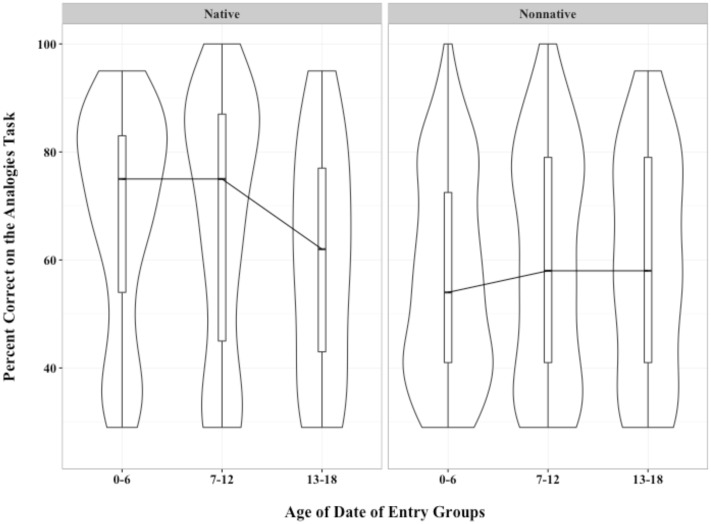
**Density plots showing percent correct on the language based analogical reasoning task for native and non-native signers by age of date of entry groups**.

To graphically depict the influence of early age of exposure on subsequent ASL syntactic and vocabulary ability, we used density plots to describe the distributions of scores, with native and non-native signers plotted in side-by-side panels (as in **Figures 2** and **3**).

The reason for using density plots is that the diversity of abilities of students in our sample meant that dividing our students into consecutive age groups results in non-normal distributions. For example, when students have only a few years in the current school of assessment (and thus lower likelihood of intensive exposure to ASL), many students cluster in the bottom of the distribution of syntactic total scores. With older ages, the distribution of ASL ability scores shifts so that an increasing proportion of students are in the middle or higher of the distribution. Density plots are a powerful method of visualizing both central tendencies and overall distribution shape. The bar in the middle of each plot is the median of the sample. The long vertical box encompassing it shows the interquartile range, similar to conventional boxplots. This box indicates the “middle fifty” in a data set. That is, when scores of a distribution are divided into fourths, the two quartiles in the middle show the middle half of the distribution. The outline of the distribution encompasses all points.

We connected each median across the three age grouping with a line, as is conventional in line graphs, to aid visualizing trends across age groupings. Medians were employed in statistical analyses because mean scores were compromised by the variability in the samples.

#### Density Plots of Syntactic Judgment Scores

As shown in **Figure 2**, for native signers, the median score on the syntactic judgment task was lowest for students with the oldest age of school entry. The distribution also changed across the three age groupings. For students who entered school between the years of 0–6, the largest bulge in the plot is around a score of 70% correct. In contrast, for students who entered school at ages 7–12, most frequent scores lie at the bottom of the distribution.

For non-native signers, median scores are almost unchanging across the age groupings. However, like the distribution for native signers, the distribution grows increasingly bottom-heavy, with half of students who entered the current school after age 12 having scores below 50% correct, indicating poor ability to select the syntactically correct sentence from the set of four options.

One question is why few students had tests scores above 80% correct (see **Figure 2**). Syntactic judgment tasks are known to be difficult. In Boudreault and Mayberry (2006), even native signers only had a mean score of 85% – and they were adults. We therefore considered 85% to the ceiling, signifying adult competence. Based on this, our best performing group, the native signers, are on an expected developmental trajectory.

#### Density Plots of Analogy Task Scores

For native signers, who entered their current school for the Deaf by age 6, analogical reasoning ability was relatively high (75% correct). Indeed, the density plots for these native signers, shown in **Figure 3**, have a bulge at 80% correct, indicating good understanding of analogies. However, scores show a different distribution for students who entered their current school after age 12. Here, 50% of the scores were lower than 60% correct.

A bleaker picture is portrayed by the distributions for non-native signers. Age of entry to the current school did not influence median scores on the analogies task. Interestingly, the age-of-school-entry group with the largest bulk of low scores was the group with the earliest age of entry. This is presumably because non-native signers with age of entry 0–6 includes young children, many of whom are just beginning to learn ASL. There is surprisingly little change from the 7–12 group to the 13–18 group. Like native signers, the non-native signers in the 13–18 group show uniform density spanning the range from 40 to 80% correct. This indicates that students with late age of school entry are a diverse group, spanning the spectrum from having poor to good analogical reasoning abilities.

## Discussion

### Overview of Findings

We investigated two variables related to age of exposure of sign language. The first variable was whether or not parents fluently signed to their children starting at birth, measured using parental hearing status (native ns. non-native). The second variable was children’s age at date of entry into an academic signing environment, which was the school for the Deaf where students were assessed. Both of these types of age-dependent language experiences influenced subsequent signing ability:

• Age-of-entry into an academic signing environment after 6 years of age was associated with poorer performance on both the syntactic judgment task and the language-based analogical reasoning task. The influence of age-of-entry was not linear. Test scores were markedly lower for Deaf children who entered the school of assessment after the age of 12.
• The positive influence of signing from birth-on was found for learners of all ages and for all age-of-entry groupings. Being a non-native signer had negative effects on later ASL skills that were similar in magnitude to entering the school for the Deaf after age 12 (i.e., see beta weights in **Table 3**).

We comment on these after describing age effects in our dataset.

### Age Effects in Syntactic Judgment and Analogical Reasoning Abilities

Age effects for the acquisition of ASL syntactic judgment abilities, documented in Novogrodsky et al. (under review), were surprisingly modest in magnitude. Effect sizes for the contribution of age to syntactic judgment scores were weaker than the effect of having Deaf parents (i.e., early exposure to language), and weaker than the cumulative number of years enrolled in a Deaf school. In addition, interpreting age effects is difficult in a naturalistic sample such as ours, because test scores can be worse for older children than for younger children. The reason is that test scores of older children include students who transferred to the current school for the Deaf after experiencing academic failure at a non-signing school. An example of this is that older children in our database included subsamples who were 10–13 years old at their first systematic exposure to ASL. For students who have been in school for the Deaf continuously, test scores are linearly correlated with age. But averaging together students who entered early with those who entered late resulted in a flattened slope for test scores as a function of age during ages 10–14.

Age at test is important because the ability to judge grammaticality is a metalinguistic ability requiring cognitive maturity. At ages 7–8, when Deaf children are able to produce syntactically correct signs, their ability on the syntactic judgment task remained poor (Novogrodsky et al., under review). At this age, non-native scorers as a group were near chance (average percent correct 27%, range 20–49%). Native signers did better, but the judgment task was still challenging, with an average percent correct of 40% (range 20–62%).

Age effects are also evident in analogical reasoning tasks that use words rather than shapes and figures (Alexander et al., 1989; Goswami, 1991; Gentner et al., 2009). Rattermann and Gentner (1998) argue that relational reasoning abilities reflect the cumulative effect of knowledge. As children age, they acquire more vocabulary and concept knowledge which helps them better detect, analyze, and apply relationships between words or concepts. The results in **Tables 2** and **3** show clear improvement in performance on the Analogies and Syntax tasks based on age. However, while age effects were present in our study, they were more limited than the effects of early exposure to language, and the cumulative effects of academic sign language in schools.

### The Advantage of Signing from Birth: Extending Prior Research

The advantages of early exposure to sign language are well documented (Mayberry and Lock, 2003; Mayberry, 2010). Our data extend these results to a large naturalistic sample of school-aged children between the ages of 7;5 to 18;6. Participants had better ASL syntax and analogical reasoning skills if they signed from birth. Native signers scored on average 13 points higher than non-native signers on an ASL syntactic judgment task, and 12 points higher on the ASL language-based analogies task. Our study is the first study to demonstrate the advantage of signing from birth on children’s ability to solve language-based analogical reasoning problems in ASL. We found high analogy test scores for Deaf students who grew up as primarily visual learners, with ASL as the native language. This refutes Sharpe’s (1985) claim that auditory stimulation is necessary to develop language-based analogical reasoning skills.

### Signing in School Facilitates ASL Syntactic Judgment and Language-Based Analogical Reasoning Abilities

Because only a small percentage of Deaf children have fluent signing experiences in the home, entry into a signing classroom represents, for most Deaf children, both the first systematic exposure to sign language and also the first exposure to academic sign language. We therefore looked at age at date of entry as another variable in the acquisition process for ASL based syntactic judgment skills and for ASL based analogical reasoning skills.

Non-native signers who entered the school of the Deaf before age 6 had better ASL abilities than did those who entered after age 6. Deaf children who entered between ages 6 and 12 had better ASL skills than those who entered after age 12 but never equaled or caught up to the native signers. These patterns held for both syntactic judgment and analogical reasoning abilities.

Native signers who entered school after the age of 12 also showed poorer performance on both syntactic judgment and analogical reasoning tasks compared to those who entered school before the age of 12. There are likely to be two reasons, which future work can investigate: as noted earlier, Deaf parents may have emphasized oral training or a different signing system at home, such as cued speech or even a different natural sign language. This will mean low levels of ASL. Another contributing factor is that sign language in the classroom is academic language. Enrollment at Deaf school may be important even for students who have ASL at home. Academic language prepares students for the metalinguistic abilities required by syntactic judgment and analogical reasoning tasks.

### Implications

In the introduction we noted two pieces of advice to hearing parents, based on quite different views of the influence of early language experiences on subsequent language development. One claim assumed that ASL acquisition is under such tight maturational constraints that hearing parents stand little chance of being able to learn it in time for children to benefit. Parents are similarly told that enrolling Deaf children in schools for the Deaf where ASL is used after early intervention periods would also be too late (Knoors and Marschark, 2012). In contrast, others have told parents to exclude sign from the home to give speech training the best chance of taking hold, with the idea that sign language could be learned later as a back-up language if oral methods failed (Mauldin, 2016). We wanted to see if the windows for learning ASL close in early childhood, or can ASL be learned well anytime in childhood provided the correct environment?

Our results demonstrate a continuum of outcomes that reflect experience with language as a continuous variable that is sensitive to maturational age. The best sign-language outcomes were for exposure from birth from parents, next best was ASL exposure before age 6, next was academic ASL exposure before 12, and the worst was academic ASL exposure after age 12. Advocates for a “windows closing” view could see their perspective supported by our findings that non-native signers as a group were delayed, at all ages tested, relative to native signers. Later school enrollment was also associated with poorer sign language than earlier entry to a signing school.

On the other hand, advocates for “sign language as a backup” could focus on the considerable overlap between native and non-native signers. As we noted, half of Deaf children with hearing parents had ASL scores which were as good as the scores achieved by three-quarters of the Deaf children with Deaf parents. This can be seen by comparing medians and quartiles across the two density plots in each of **Figures 2** and **3**. Comparing native and non-native signers showed the robust advantage of being a native signer, but the density charts also demonstrate that many Deaf children with hearing parents performed as well as did their native-signing peers. Finally, some non-native signers who enrolled after age 12 also were able to score on our tasks at similar levels to native signers. These children may have had some signing experience before transferring into the school of assessment. Ultimately, the results show that time spent in a good signing environment (e.g., ASL based programs for the Deaf) leads to across the board improvements in language and language related abilities in Deaf children.

We suggest that the best advice for parents is to avoid all-or-none thinking, and recognize the impact of the continuum of age-related declines revealed by our data. The best signing outcome is when signing occurs from birth, but considerable plasticity exists and most students may be able to take advantage of the fully accessible exposure to ASL when presented consistently by peers and adults. Our advice is that parents should, when possible, choose ASL as either a primary or a supplementary means of communicating with their Deaf children.

## Conclusion

Our findings confirm and extend to a large naturalistic sample the well-known advantage of early, systematic exposure to ASL. Native signers had an advantage in ASL syntactic skills and vocabulary-based analogical reasoning that held irrespective of age-of-entry to an academic signing environment. While native signers will remain a small percent of the Deaf population, this shows that hearing parents who learn to sign can and do influence their children’s language skills and the development of their higher level language abilities. We additionally documented that non-native signers have the best chance of developing their ASL abilities when they are exposed to an academic signing environment before the age of 12. Parents who place their children in good signing programs for the Deaf by age 6 (for the best result) can expect that their children will approximate the language skills of native-signing, Deaf children.

## Notes

The data used in this analysis has also been used in other analyses by the same team. However, we affirm that the analysis and discussion here is novel.

## Ethics Statement

IRB approval was provided by the Boston University Charles River Campus Institutional Review Board. Consent for data collection was provided via Blanket Consent procedures. Parents were required to opt their children out of assessment. Information about the assessment was provided in both print and via ASL videos. Adults over the age of 18 who were included in assessment were also provided text or video consent documents.

## Author Contributions

JH was the lead writer for this article. He did the analyses and guided the team during the writing process. CC-H was vital during the editing process. She helped make the article appropriate for publication in Frontiers. RN helped JH build the initial few drafts and developed the theoretical background. RH developed the application that collected the data, built the research team, and helped edit the draft.

## Conflict of Interest Statement

The authors declare that the research was conducted in the absence of any commercial or financial relationships that could be construed as a potential conflict of interest.

## References

[B1] Amraei K. Amirsalari S. Ajalloueyan M. (2017). Comparison of intelligence quotients of first- and second-generation deaf children with cochlear implants. *Int. J. Pediatr. Otorhinolaryngol.* 92 167–170. 10.1016/j.ijporl.2016.10.005 28012522

[B2] Alexander P. A. Willson V. L. White C. S. (1989). Development of analogical reasoning in 4-and 5-year-old children. *Cogn. Dev.* 4 65–88. 10.1016/0885-2014(89)90005-1

[B3] Anderson M. L. Glickman N. S. Mistler L. A. Gonzalez M. (2015). Working therapeutically with deaf people recovering from trauma and addiction. *Psychiatr. Rehabil. J.* 39 27–32. 10.1037/prj0000146 25984736 PMC4651859

[B4] Andrew K. N. Hoshooley J. Joanisse M. F. (2014). Sign language ability in young deaf signers predicts comprehension of written sentences in english. *PLoS ONE* 9:e89994 10.1371/journal.pone.0089994 PMC3938551 24587174

[B5] Archibold S. Nikolopoulous T. Lloyd-Richmond H. (2009). Long term use of cochlear implant systems in paedriati recipients and factors contributing to non-use. *Cochlear Implants Int.* 10 25–40. 10.1002/cii.363 18979457

[B6] Ausbrooks M. M. Gentry M. A. Martin G. (2014). Exploring linguistic interdependence between American sign language and english through correlational and multiple regression analyses of the abilities of biliterate deaf adults. *Int. J. Engl. Linguist.* 4 1–18.

[B7] Baayen R. H. Davidson D. J. Bates D. M. (2008). Mixed-effects modeling with crossed random effects for subjects and items. *J. Mem. Lang.* 59 390–412. 10.1016/j.jml.2007.12.005

[B8] Bandurski M. Galkowski T. (2004). The development of analogical reasoning in deaf children and their parents’ communication mode. *J. Deaf Stud. Deaf Educ.* 9 153–175. 10.1093/deafed/enh018 15304438

[B9] Black P. A. Glickman N. S. (2006). Demographics, psychiatric diagnoses, and other characteristics of North American deaf and hard-of-hearing inpatients. *J. Deaf Stud. Deaf Educ.* 11 303–321. 10.1093/deafed/enj042 16687730

[B10] Boudreault P. Mayberry R. I. (2006). Grammatical processing in American sign language: age of first-language acquisition effects in relation to syntactic structure. *Lang. Cogn. Process.* 21 608–635. 10.1080/01690960500139363

[B11] Branson J. Miller D. (1993). Sign language, the deaf and the epistemic violence of mainstreaming. *Lang. Educ.* 7 21–41. 10.1080/09500789309541346

[B12] Corina D. Singleton J. (2009). Developmental social cognitive neuroscience: insights from deafness. *Child Dev.* 80 952–967. 10.1111/j.1467-8624.2009.01310.x 19630887

[B13] Cummins J. (1982). *Tests, Achievement, and Bilingual Students. Focus.* Wheaton, MD: National Clearinghouse for Bilingual Education, 9.

[B14] Curtiss S. (1977). *Genie: A Psycholinguistic Study of a Modern-day “Wild Child.”.* Cambridge: Academic Press, Inc.

[B15] Davidson K. Lillo-Martin D. Pichler D. C. (2014). Spoken english language development among native signing children with cochlear implants. *J. Deaf Stud. Deaf Educ.* 19 238–250. 10.1093/deafed/ent045 24150489 PMC3952677

[B16] Edwards L. Figueras B. Mellanby J. Langdon D. (2011). Verbal and spatial analogical reasoning in deaf and hearing children: the role of grammar and vocabulary. *J. Deaf Stud. Deaf Educ.* 16 189–197. 10.1093/deafed/enq051 21068179

[B17] Figueras B. Edwards L. Langdon D. (2008). Executive function and language in deaf children. *J. Deaf Stud. Deaf Educ.* 13 362–377. 10.1093/deafed/enm067 18252699

[B18] Gallaudet Research Institute (2011). *Regional and National Summary Report of Data from the 2009–2010 Annual Survey of Deaf and Hard of Hearing Children and Youth.* Washington, DC: Gallaudet University Press.

[B19] Geers A. Nichols J. Tobey E. Davidson L. (2015). Language emergence in early-implanted children. *Acoust. Soc. Am.* 137 2015 10.1121/1.4920258

[B20] Geers A. Sedey A. (2011). Language and verbal reasoning skills in adolescents with 10 or more years of cochlear implant experience. *Ear Hear.* 32 1–19. 10.1097/AUD.0b013e3181fa41dc PMC3157037 21832889

[B21] Gentner D. Simms N. Flusberg S. (2009). “Relational language helps children reason analogically,” in *Proceedings of the 31st Annual Conference of the Cognitive Science Society*, London, 1054–1059.

[B22] Giraud A.-L. Lee H.-J. (2007). Predicting cochlear implant outcome from brain organisation in the deaf. *Restor. Neurol. Neurosci.* 25 381–390. 17943013

[B23] Glickman N. (2007). Do you hear voices? problems in assessment of mental status in deaf persons with severe language deprivation. *J. Deaf Stud. Deaf Educ.* 12 127–147. 10.1093/deafed/enm001 17290050

[B24] Goldin-Meadow S. Mylander C. (1998). Spontaneous sign systems created by deaf children in two cultures. *Nature* 391 279–281. 10.1038/34646 9440690

[B25] Goswami U. (1991). Analogical reasoning: what develops? A review of research and theory. *Child Dev.* 62 1–22. 10.2307/1130701

[B26] Hassanzadeh S. (2012). Outcomes of cochlear implantation in deaf children of deaf parents: comparative study. *J. Laryngol. Otol.* 126 989–994. 10.1017/S0022215112001909 22906641

[B27] Henner J. (2016). *The Relationship Between American Sign Language Vocabulary and the Development of Language-Based Reasoning Skills in Deaf Children.* Doctoral Dissertation, Boston University, Boston, MA.

[B28] Henner J. Hoffmeister R. Fish S. Rosenburg P. DiDonna D. (2015). Bilingual instruction works even for deaf children of hearing parents. *Paper Presented at the 2015 American Educational Research Association*, Chicago, IL.

[B29] Hrastinski I. Wilbur R. B. (2016). Academic achievement of deaf and hard-of-hearing students in an ASL/English bilingual program. *J. Deaf Stud. Deaf Educ.* 21 156–170. 10.1093/deafed/env072 26864688 PMC4886322

[B30] Knoors H. Marschark M. (2012). Language planning for the 21st century: revisiting bilingual language policy for deaf children. *J. Deaf Stud. Deaf Educ.* 17 291–305. 10.1093/deafed/ens018 22577073

[B31] Kronenberger W. G. Pisoni D. B. Henning S. C. Colson B. G. (2013). Executive functioning skills in long-term users of cochlear implants: a case control study. *J. Pediatr. Psychol.* 38 902–914. 10.1093/jpepsy/jst034 23699747 PMC3747713

[B32] Lange C. M. Lane-Outlaw S. Lange W. E. Sherwood D. L. (2013). American sign language/english bilingual model: a longitudinal study of academic growth. *J. Deaf Stud. Deaf Educ.* 18 532–544. 10.1093/deafed/ent027 23741074

[B33] Mauldin L. (2016). *Made to Hear: Cochlear Implants and Raising Deaf Children.* Minneapolis, MN: University of Minnesota Press.

[B34] Mayberry R. (1993). First-language acquisition after childhood differs from second-language acquisition: the case of American sign language. *J. Speech Hear. Res.* 36 1258–1270. 10.1044/jshr.3606.1258 8114493

[B35] Mayberry R. (2007). When timing is everything: age of first-language acquisition effects on second-language learning. *Appl. Psycholinguist.* 28 537–549. 10.1017/S0142716407070294

[B36] Mayberry R. (2010). “Early language acquisition and adult language ability: what sign language reveals about the critical,” in *The Oxford Handbook of Deaf Studies, Language, and Education* Vol. 2 eds Marschark M. Spencer P. E. (Oxford: Oxford University Press), 281–291.

[B37] Mayberry R. Chen J.-K. Witcher P. Klein D. (2011). Age of acquisition effects on the functional organization of language in the adult brain. *Brain Lang.* 119 16–29. 10.1016/j.bandl.2011.05.007 21705060

[B38] Mayberry R. Eichen E. (1991). The long-lasting advantage of learning sign language in childhood: another look at the critical period for language acquisition. *J. Mem. Lang.* 30 486–512. 10.1016/0749-596X(91)90018-F

[B39] Mayberry R. Fischer S. (1989). Looking through the phonological shape to lexical meaning: the bottleneck of non-native sign language processing. *Mem. Cogn.* 17 740–754. 10.3758/BF03202635 2811671

[B40] Mayberry R. Lock E. (2003). Age constraints on first versus second language acquisition: evidence for linguistic plasticity and epigenesis. *Brain Lang.* 87 369–384. 10.1016/S0093-934X(03)00137-8 14642540

[B41] Mayberry R. Lock E. Kazmi H. (2002). Linguistic ability and early language exposure. *Nature* 417 38 10.1038/417038a 11986658

[B42] Mayberry R. Squires B. (2006). “Sign language acquisition,” in *Encyclopedia of Language & Linguistics*, 2nd Edn Vol. 11 ed. Brown K. (Oxford: Elsevier), 291–296.

[B43] Mayberry R. I. (1992). “The cognitive development in deaf children: recent insights,” in *Handbook of Neuropsychology* Vol. 8 eds Segalowitz S. J. Rapin I. (Amsterdam: Elsevier), 71–107.

[B44] Mellon N. K. Niparko J. K. Rathmann C. Mathur G. Humphries T. Napoli D. J. (2015). Should All deaf children learn sign language? *Pediatrics* 136 170–176. 10.1542/peds.2014-1632 26077481

[B45] Mitchell R. E. Karchmer M. A. (2005). Parental hearing status and signing among deaf and hard of hearing students. *Sign Lang. Stud.* 5 231–244. 10.1353/sls.2005.0004

[B46] Newport E. (1990). Maturational constraints on language learning. *Cogn. Sci.* 14 11–28. 10.1207/s15516709cog1401_2

[B47] Newport E. Meier R. P. (1985). “The acquisition of american sign language,” in *The Cross-Linguistic Study of Language Acquisition*, ed. Slobin D. I. (Hillsdale, NJ: Erlbaum), 881–938.

[B48] Niparko J. K. Tobey E. A. Thal D. J. Eisenberg L. S. Wang N. Y. Quittner A. L. (2010). Spoken language development in children following cochlear implantation. *JAMA* 303 1498–1506. 10.1001/jama.2010.451 20407059 PMC3073449

[B49] Nishimura H. Hashikawa K. Doi K. Iwaki T. Watanabe Y. Kusuoka H. (1999). Sign language “heard”in the auditory cortex. *Nature* 397 116 10.1038/16376 9923672

[B50] Novogrodsky R. Caldwell-Harris C. Fish S. Hoffmeister R. J. (2014a). The development of antonym knowledge in American Sign Language (ASL) and its relationship to reading comprehension in English. *Lang. learn.* 64 749–770. 10.1111/lang.12078

[B51] Novogrodsky R. Fish S. Hoffmeister R. (2014b). The acquisition of synonyms in American Sign Language (ASL): toward a further understanding of the components of ASL vocabulary knowledge. *Sign Lang. Stud.* 14 225–249. 10.1353/sls.2014.0003

[B52] Nunes T. Pretzlik U. Olsson J. (2001). Deaf children’s social relationships in mainstream schools. *Deafness Educ. Int.* 3 123–136. 10.1179/146431501790560972

[B53] Pénicaud S. Klein D. Zatorre R. J. Chen J.-K. Witcher P. Hyde K. (2013). Structural brain changes linked to delayed first language acquisition in congenitally deaf individuals. *Neuroimage* 66 42–49. 10.1016/j.neuroimage.2012.09.076 23063844

[B54] Petitto L. A. (1987). On the autonomy of language and gesture: evidence from the acquisition of personal pronouns in american sign language. *Cognition* 27 1–52. 10.1016/0010-0277(87)90034-5 3691016

[B55] Ramsey C. (1997). *Deaf Children in Public Schools: Placement, Context, and Consequences.* Washington, DC: Gallaudet University Press.

[B56] Rattermann M. J. Gentner D. (1998). More evidence for a relational shift in the development of analogy: children’s performance on a causal-mapping task. *Cogn. Dev.* 13 453–478. 10.1016/S0885-2014(98)90003-X

[B57] Richland L. E. Burchinal M. R. (2013). Early executive function predicts reasoning development. *Psychol. Sci.* 24 87–92. 10.1177/0956797612450883 23184588

[B58] Sharpe S. (1985). The primary mode of human communication and complex cognition. *Am. Ann. Deaf* 130 39–46. 10.1353/aad.2012.0928 3993507

[B59] Singleton D. Ryan L. (2004). *Language Acquisition: The Age Factor.* Bristol: Multilingual Matters.

[B60] Watson L. M. Gregory S. (2005). Non-use of cochlear implants in children: child and parent perspectives. *Deafness Educ. Int.* 7 43–58. 10.1179/146431505790560482

[B61] Williams K. (2007). *EVT-2: Expressive Vocabulary Test.* Minneapolis, MN: Pearson Assessments.

[B62] Yoshinaga-Itano C. Baca R. Sedey A. (2010). Describing trajectory of language development in the presence of severe to profound hearing loss: a closer look at children with cochlear implants versus hearing aids. *Otol. Neurotol.* 31 1268–1274. 10.1097/MAO.0b013e3181f1ce07 20818291 PMC3014847

[B63] Zimmerman I. Steiner V. Pond R. (2002). *Preschool Language Scale.* San Antonio, TX: Psychological Corporation.

